# Embedding Assessment Literacy Can Enhance Graduate Attribute Development in a Biomedical Sciences Curriculum

**DOI:** 10.3389/bjbs.2024.12229

**Published:** 2024-05-24

**Authors:** Kevin A. Robertson, Kirsty J. Hughes, Susan M. Rhind

**Affiliations:** ^1^ Infection Medicine, Biomedical Teaching Organisation, Deanery of Biomedical Sciences, The University of Edinburgh, Edinburgh, United Kingdom; ^2^ Veterinary Medical Education, Royal (Dick) School of Veterinary Studies, University of Edinburgh, Edinburgh, United Kingdom

**Keywords:** assessment literacy, graduate attributes, biomedical sciences, feedback, peer assessment

## Abstract

This paper describes the successful implementation of an assessment literacy strategy within a Biomedical Sciences degree. Teaching was aligned with an assessment literacy framework and aimed to prepare undergraduates for a literature comprehension assessment. Students were introduced to the assessment purpose and an adapted Miller’s pyramid model illustrated how the assessment contributed to competency development during their degree. Students read primary research papers and answered questions relating to the publications. They were then introduced to the processes of assessment and collaboratively graded answers of different standards. Finally, student and faculty grades were compared, differences considered, and key characteristics of answers discussed. Most students reported that they understood more about assessment standards than prior to the intervention [139/159 (87.4%)] and felt it had helped prepare them for their exam [138/159 (86.8%)]. The majority also reported they had increased confidence in evaluating data [118/159 (74%)], communicating their reasoning [113/159 (71%)] and considering what a reader needs to know [127/159 (79.9%)]. Students were asked to state the most important thing they had learned from the assessment literacy teaching. Notably, no responses referred to domain-specific knowledge. 129 free text responses were mapped to the University of Edinburgh graduate attribute framework. 93 (72%) statements mapped to the graduate attribute category “Research and Enquiry,” 66 (51.16%) mapped to “Communication” and 21 (16.27%) mapped to “Personal and Intellectual Autonomy.” To explore any longer-term impact of the assessment literacy teaching, a focus group was held with students from the same cohort, 2 years after the original intervention. Themes from this part of the study included that teaching had provided insights into standards and expectations for the assessment and the benefits of domain specific knowledge. A variety of aspects related to graduate attributes were also identified. Here, assessment literacy as a vehicle for graduate attribute development was an unexpected outcome. We propose that by explicitly engaging students with purpose, process, standards, and expectations, assessment literacy strategies may be used to successfully raise awareness of developmental progression, and enhance skills, aptitudes, and dispositions beneficial to Biomedical Sciences academic achievement *and* life after university.

## Introduction

Undergraduate Biomedical Sciences (BMS) degree programmes typically provide an interdisciplinary context in which learning about the science underpinning human health and disease is enabled [1]. Importantly, alongside domain-specific learning, it is now widely accepted that higher education should prepare graduates for work and life after their formal studies [2]. In this regard, BMS degrees are no different to any other. Over the past two decades, increasing numbers of fee-paying students, with broad career aspirations, and often significant debt, have created demand for the development of employability during a first degree [2, 3]. BMS programme developers have responded to this in a variety of ways. Examples include the placement of students with employers, the delivery of employability workshops and/or an increased emphasis on integrating opportunities to enhance competency development and graduate attributes within curricula [4–6]. Generic graduate attributes include, for example, competency in reflective practice, communication with diverse audiences, complex problem solving, assessing the performance of self and others, an inclusive and open attitude to engaging with others and intellectual autonomy [7, 8]. A consequence of approaches targeted at integrating domain-specific and generic competencies can be curriculum complexity. This can make it challenging for students to navigate and understand their developmental progression.

Confidence in reading, analysing, interpreting, presenting, and using primary evidence to learn, develop hypotheses, solve problems, and enable decision-making (i.e., “literature comprehension”) is integral to all research practice. It is also a health and care professions council (HCPC) requirement for Biomedical Scientists and is key to many graduate careers [9]. Competency in literature comprehension is, therefore, considered a core graduate attribute for all BMS graduates. At the University of Edinburgh (UoE), the BMS Literature Comprehension assessment (LCA) serves as an introduction for a diverse cohort of several hundred 2nd year undergraduates per year to the critical analysis of primary research. At this early stage, it is intended to facilitate the transition of students into their degree (and enhance inclusivity) by (a) clarifying expectations on how practising scientists analyse and use primary research material and (b) delivering a common understanding of standards and expectations prior to summative testing [10].

Since its inception in the early 2000s, the LCA has involved two formative tutorials and an open-book exam. Across the teaching and assessment, students analyse multiple primary research papers in-depth. By responding to short answer questions related to these papers, it is hoped participants can develop their approach to analysing primary evidence and communicating their own interpretations in a concise, logical manner. Before students attempt the summative assessment, they have extensive opportunities to develop their learning—key to both assessment *for* learning and inclusivity [10, 11]. The literature comprehension assessment is not a test of memory, rather it presents an authentic challenge relevant to careers in BMS. In this regard, it serves to develop several attributes considered key by the Institute of Biomedical Science (IBMS). For example, questions require that students explain their rationale and use data to support conclusions. As such, the assessment establishes a foundation for biomedical competencies such as the communication of research findings using appropriate scientific language [1]. End of course feedback from students has described the LCA as *“challenging yet rewarding”* and an opportunity to *“feel like a scientist*.” Importantly, integrated within the domain-specific teaching of the LCA are also opportunities for students to develop (a) a general framework for thinking about evidence and (b) how they communicate to different audiences—both crucial to graduate attributes such as a capacity for critical/analytical thinking and ability to communicate in a variety of contexts [1].

**FIGURE 1:**
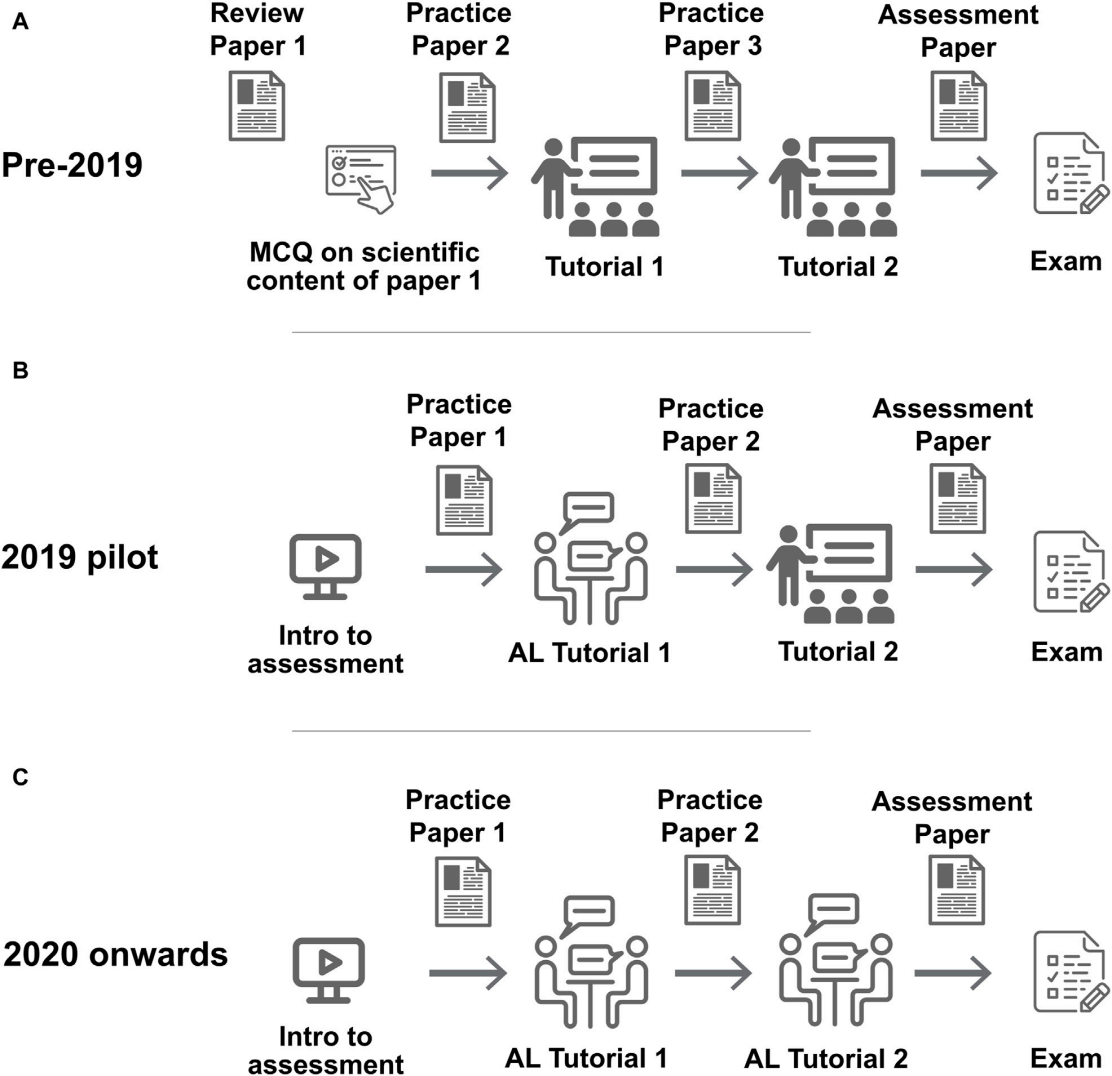
Delivery of literature comprehension teaching before and after the assessment literacy intervention. **(A)** Literature comprehension teaching prior to the assessment literacy intervention. Students engaged with scientific material in Review paper 1 by reading the publication and answering online multiple-choice questions focused on the scientific content of the review. Students then participated in two teacher-led tutorials intended to prepare them for a subsequent assessment paper. Students read a paper then answered questions prior to each session. In the sessions, tutors would lead students through the study and endeavour to generate discussion by, for example, asking students to share their answers. **(B)** Pilot assessment literacy-based literature comprehension intervention. A brief online recorded presentation replaced the first review paper activity and introduces students to the teaching approach and purpose of the assessment. Prior to tutorial 1, students read a primary research paper and answer questions related to the publication. In a revised tutorial 1, students work collaboratively to grade authentic answers with the assessment marking scheme. After grading, group marks are compared with those assigned by faculty. To conclude, tutors and students discuss the question “What makes a good answer?”. Tutors then review answers of different standards, facilitate a discussion on key features that are rewarded and discuss the scientific content of the paper. Tutorial 2 is delivered as in previous years. **(C)** The assessment literacy-based teaching described for tutorial 1 above is implemented in both tutorials.

Prior to 2019, the LCA was delivered at the UoE as shown in Figure 1A. At this time, a course review identified a range of issues related to teaching and assessment that needed to be addressed. These were (a) uncertainty in the student cohort regarding the purpose of the exercise (b) a tutor-focused teaching approach leading to inconsistent engagement of students in tutorials (c) inconsistent student communication of thinking and rationale in exams (d) inconsistent use of data/evidence to support answers in exams and (e) students regularly reporting that they felt, “*the exam was much harder than the tutorial exercises.*” To address these issues, an intervention focused on assessment literacy was identified as a potential solution.

**FIGURE 2:**
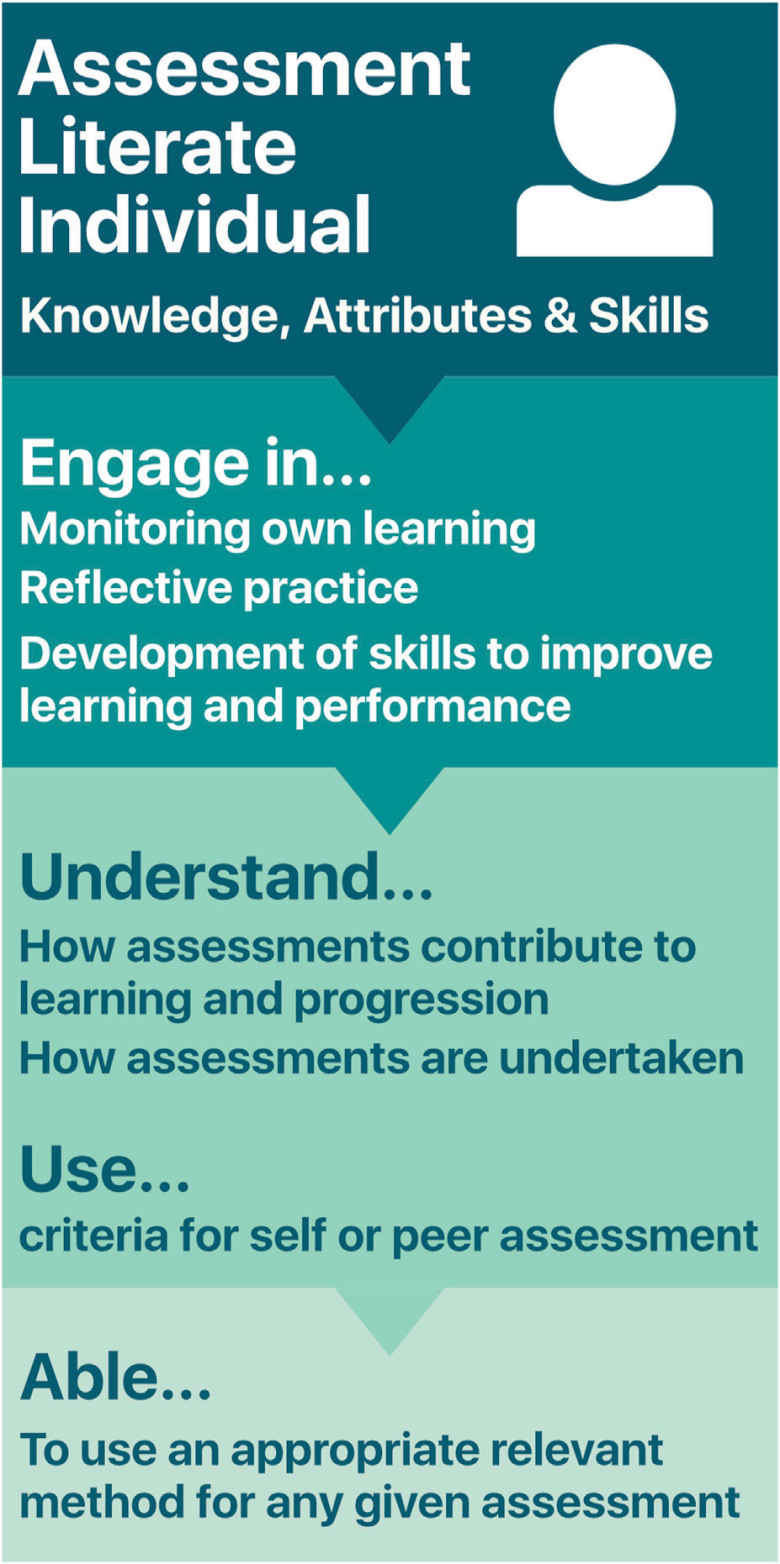
Characteristics of an assessment literate individual.

The concept and benefits of assessment literacy have been widely discussed [12–16]. In this regard, a recent review has comprehensively defined a conceptualisation defining domains and dispositions required by students to engage with assessment in an effective manner [16]. In brief, an assessment literate individual has the knowledge, attributes, and skills to “actively engage in assessment, monitor their learning, engage in reflective practice, and develop effective skills, to improve their learning and performance outcomes” (Figure 2) [16]. Further, they will understand how assessments contribute to learning and progression, how assessments are undertaken and can use criteria for self or peer assessment. Given this understanding, an assessment literate student will be able to use an appropriate, relevant method for any given assessment task [13]. Crucially, an absence of assessment literacy can impede an individual’s capacity to learn and, if assessment literacy is not promoted, it can limit inclusivity, equity and participation in higher education [15].

In 2015, an assessment literacy intervention was used to enhance veterinary undergraduate teaching at the UoE [13]. In this intervention, the use of Miller’s pyramid helped promote a common understanding (in teachers *and* students) of curriculum progression and, importantly, how a given specific assessment functioned within the curriculum. Miller’s pyramid has been widely used as a model for assessing levels of clinical competence [17, 18]. In the pyramid, cognitive levels “knowledge” (“Knows”) and “application of knowledge” (“Knows how”) function as a foundation for a subsequent “practical application of knowledge” (“Shows how”) which in turn supports “Does”—representing (graduate) practitioner competence. Notably, the 2015 intervention required that students evaluate authentic work of differing quality and discuss attributes that are valued by learners and staff. This resulted in a better understanding of standards, and helped students prepare for a subsequent assessment [13, 18]. Given the success of this assessment literacy intervention, a novel Assessment Literacy Pyramid (ALP) designed to support student assessment of their own and peer performance at all levels of a developmental programme has subsequently been developed [18].

The primary aim of this study was to evaluate assessment literacy as a unifying concept and practical approach to enhance literature comprehension in the context of a BMS curriculum. Specifically, the objective was to explore whether assessment literacy could; clarify for students why an assessment was being used, clarify expectations regarding assessment criteria, answer questions, address past criticisms, improve engagement in, and inclusivity of, teaching sessions, enhance student capacity for self-evaluation and, ultimately, make the assessment less intimidating. As part of this work, we aimed to develop a BMS competency pyramid to enhance communication of curriculum opportunities and progression to our students.

## Methods

### Teaching Context

This study was undertaken with students in the 2nd year (Scottish Credit and Qualifications Framework Level 8) of a 4-year non IBMS accredited BMS degree programme. The literature comprehension assessment was a component of a single semester compulsory course focused on the fundamentals of infection and immunity (Learning outcomes presented in Supplementary Table S1). Students were required to pass all components (exam, essay, and literature comprehension assessment) of the course to progress to the next academic year. As per standard UoE practice, a range of adjustments were provided to students according to individualised profiles developed by the student and the university Disability and Learning Support Service (DLSS). Adjustments included, for example, extra time for submission of the assessment and the provision of time for students to use proof-reading services. Additionally, for use with screen readers and to enable reformatting, accessible versions of primary research papers (converted to plain HTML, with ALT tag descriptions of data and validated by staff in the DLSS) were available.

Prior to and including 2018, teaching related to the literature comprehension assessment was as shown in Figure 1A. In brief, all students read three papers (one per week over a 3-week period) prior to undertaking their assessment. After reading review paper 1, students answered online multiple-choice questions related to the scientific detail of the study. For papers 2 and 3, students read the primary research publications and then answered short-answer questions related to the paper. They then attended tutor-driven teaching sessions in which staff led students through the paper, and students were invited to discuss and report back on their answers. Students were provided with a primary research paper 1 week before their exam. For the 90-min exam, students were permitted to use an annotated copy of the paper to help them answer 12 to 14 short answer questions of a similar style to those they had previously encountered in the formative work.

### Assessment Literacy Intervention

To test whether an assessment literacy-based teaching approach could address the issues encountered prior to 2019 (detailed in the introduction), a phased assessment literacy intervention was designed based on previous work [13]. The development of this intervention is presented in Figures 1B, C.

#### Phase 1 of Intervention (2019)

**FIGURE 3:**
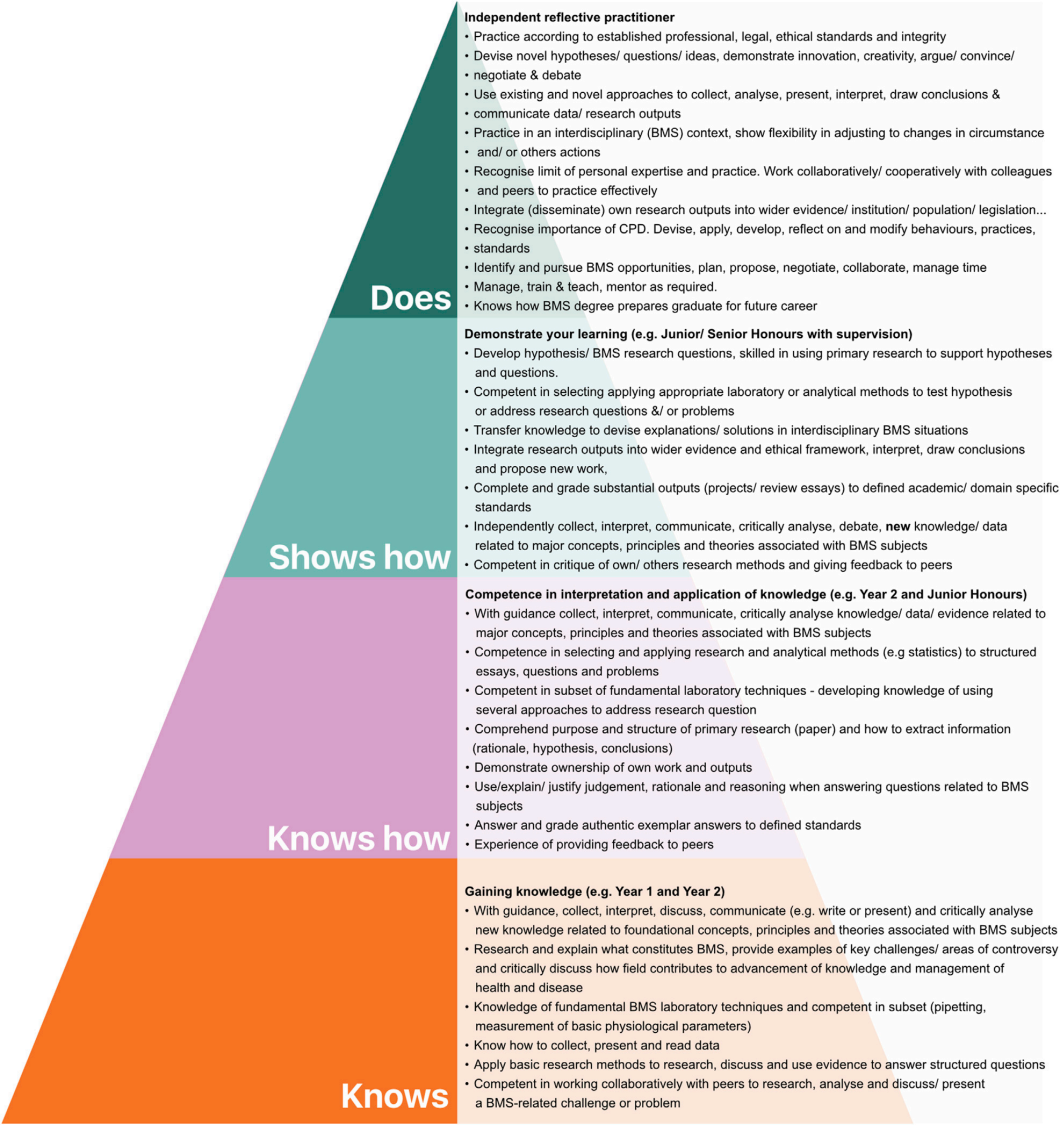
Biomedical Sciences: Undergraduate to Practitioner Competency Pyramid. Using Miller’s pyramid as a framework, the UoE degree finder (2022-2023) and the Subject Benchmark Statement for Biomedical Scientists (2019) were used to identify and map competency development from degree entry to reflective practitioner.

In phase 1 of the intervention (Figure 1B), Review paper 1 and the associated MCQ were replaced with a brief pre-recorded presentation (available in Supplementary Material) designed to introduce the purpose of the assessment and address questions often asked about the teaching material. Notably, as part of this intervention, a BMS competency pyramid (based on Miller’s pyramid) was developed to help convey and define the function of the assessment in the BMS curriculum. In recent years, Miller’s pyramid (and adaptations of the model) have been successfully used as an integral component of assessment literacy interventions [13, 18]. In this context, it can show students (a) where they are in their competency development and (b) what function the assessment literacy intervention will play in their development of new competencies. It was hoped the BMS competency pyramid would serve as a useful tool for representing the bridge between academic degree learning and graduate practice. To build a pyramid model with a BMS focus, two main resources were used to identify desirable competencies for each level. Firstly, the UoE degree finder was used to define year-on-year development of BMS knowledge, skills and attributes. Alongside this, desirable competencies drawn from the Subject Benchmark Statement for BMS were also integrated into the pyramid model at all levels [19]. Supplementary Figure S1 illustrates how early stages of the BMS model evolved from Miller’s pyramid to the integration of a preliminary subset of attributes and competencies broadly related to literature comprehension. The current BMS competency pyramid is presented in Figure 3.

The first tutor-led teaching session was also adjusted in phase 1 (2019) of our assessment literacy intervention (Figure 1B). In the new tutorial, students were introduced to the processes of assessment and the benefits of the assessment to competency development were discussed. Most importantly, students then worked together to grade authentic answers of different standards from previous years. To conclude, student grades were collated and compared with those of faculty and exemplar answers were analysed and discussed to identify characteristics that were rewarded during the marking process. A representative example of a question, analysis of student responses and marking criteria are presented in the Supplementary Material (Supplementary Figure S2). Following the 2019 pilot intervention, feedback on revised teaching was gathered as part of the standard deanery-wide end of course survey. In this survey, all students were invited to complete an electronic feedback form that included eight tutorial-focused Likert scale questions and a free text question in which respondents were asked to provide comments on the tutorial teaching and associated assessment (Supplementary Table S2).

#### Phase 2 of Intervention (2020)

In 2020, all LCA teaching was migrated to the assessment literacy-based approach (Figure 1C). All students were provided with an introductory presentation followed by two tutorials in which they graded authentic answers using a marking scheme, compared marks with those of faculty and discussed desirable features of an answer (as described above). To analyse the effects of our 2020 teaching (completed before disruption due to the COVID pandemic), a short paper-based survey was distributed to 186 students at the conclusion of tutorial 2. This questionnaire was intended to explore student expectations and understanding of assessment and whether students felt prepared for the literature comprehension test. Notably, this survey was also used to analyse student opinions on the importance of graduate attribute development and their awareness of how and when they are developing graduate attributes. Survey questions are presented in Tables 1, 2. Students were presented with 12 statements about assessment or graduate attributes and asked to indicate their level of agreement with these statements on a 5-point Likert scale from strongly disagree to strongly agree. Responses to 2 free text questions were also captured. Free text questions asked students to (a) “give examples of graduate attributes you think you have already developed as part of your studies at the University of Edinburgh?” And (b) “state the most important thing you learned from the literature comprehension tutorials.”

**TABLE 1:** Positive impact of assessment literacy intervention on student confidence in literature comprehension assessment. Year 2 Biomedical Sciences students who had completed the literature comprehension assessment tutorials in 2020 were asked to respond to nine statements related to their understanding of assessment and the outcomes of the assessment literacy tutorial teaching. Table shows 159 responses recorded using a Likert scale as follows: Strongly Disagree (SD), Disagree (D), No Strong Feelings (NSF), Agree (A), Strongly Agree (SA), Not Applicable (N/A).

Question	SD	D	NSF	A	SA	N/A	Total
I have a good understanding of how my assessments have been marked up to this point in my degree	1	14	32	78	33	1	159
I don’t think it is necessary to understand how our assessments are marked	126	21	5	4	2	1	159
The Literature Comprehension tutorials helped me understand more about different standards in assessment	0	0	18	65	74	2	159
The Literature Comprehension tutorials helped me understand how to prepare for the literature comprehension exam	1	1	17	69	69	2	159
The Literature Comprehension Tutorials helped me feel more confident in communicating my scientific interpretation and reasoning	1	9	34	77	36	2	159
The Literature Comprehension Tutorials have made me consider what a reader needs to know	0	8	22	69	58	2	159
The Literature Comprehension Tutorials have helped me understand how to evaluate and use data to support my interpretation	1	5	33	76	42	2	159
I enjoyed the literature comprehension tutorials	3	7	36	83	28	2	159
I would like similar tutorials in my other courses	2	16	21	63	55	2	159

**TABLE 2:** The development of graduate attributes is highly valued by undergraduates. Year 2 Biomedical Sciences students who had completed the literature comprehension assessment tutorials in 2020 were asked to respond to three statements related to graduate attribute development in their degree. Table presents data from 159 responses recorded using a Likert scale as follows: Strongly disagree (SD), Disagree (D), No Strong Feelings (NSF), Agree (A), Strongly Agree (SA), Not Applicable (N/A).

Statement	SD	D	NSF	A	SA	N/A	Total
The development of graduate attributes is an important part of my degree	0	0	12	59	88	0	159
I know when teaching activities are contributing to the development of my graduate attributes	4	9	49	72	25	0	159
I don’t think it is important for me to understand how graduate attributes are developed	86	57	8	6	2	0	159

### Assessment Literacy Intervention: Data Collection, Processing, and Analysis

#### Student and Faculty Grading Data

Grades awarded by students to each of five questions were recorded in eight tutorials undertaken in 2020. To explore the accuracy of student grading in relation to the faculty grade, student bias was calculated as an average of the difference between each student grade and the recorded faculty grade for each question. The percentage bias as a function of the actual grade for each question was then calculated. This provides a measure of how the mean of the student grades relates to the faculty grade. The root mean square error (RMSE) was also calculated to reflect the variation of student grades around the faculty grade (i.e., it provides a descriptive evaluation of the differences between the faculty grade and the student grades).

#### Assessment Literacy Questionnaire Data Processing and Analysis

Likert scale data from 159/186 questionnaires returned (85% response rate) in 2020 were compiled and, for each question, the total number of responses for each of the 5 options [strongly disagree (SD), disagree (D), no strong feelings (NSF), agree (A) or strongly agree (SA)] was calculated and tabulated.

#### Analysis of Free Text Responses to Graduate Attribute Development and Learning

Free text responses to the questions (a) “give examples of graduate attributes you think you have already developed as part of your studies at the University of Edinburgh?” And (b) “state the most important thing you learned from the literature comprehension tutorials” were mapped to UoE graduate attributes [19]. In brief, 115 free text responses to the question “Can you give examples of graduate attributes you think you have already developed as part of your studies at the University of Edinburgh?” were compiled. Each of the responses was then classified according to whether they represented “Mindset” and/or a “Skill Group” as defined in the UoE framework for graduate attributes (summarised in Supplementary Figure S3) [19]. Where possible, each response was further classified according to one or more sub skill groups (e.g., Research and Enquiry [Analytical Thinking]). Classifications were not mutually exclusive, and one statement could be assigned several headings. During this process, 18 responses were excluded from further analysis where the meaning of the written response was unclear/ambiguous (Supplementary Table S3).

129 free text responses to the question “state the most important thing you learned from the literature comprehension tutorials” were analysed in an identical manner to that described above. During this process, 13 responses were excluded from further analysis where the meaning of the written response was unclear/ambiguous (Supplementary Table S4).

### Focus Group Analysis of Long-Term Intervention Impact

In 2022, to explore the long-term impact of the 2020 assessment literacy teaching, final year students who had experienced the intervention (*n* = 186) were sent an open invitation by email to contribute to a focus group. Four students responded to the invitation. Having read a further information form and provided their written consent, the 4 students attended an online focus group lasting roughly 1 h. The focus group was facilitated by a UoE academic with no BMS teaching involvement who sought to gather student feedback on (amongst other aspects) recollections of the LCA purpose, opinions on how it helped their ability to use primary papers, how teaching helped understanding of assessment process and the broader impacts of the teaching. Focus group questions are presented in Table 3.

**TABLE 3:** Questions used in focus groups intended to analyse long term impact of assessment literacy intervention.

Questions regarding literature comprehension tutorials
1. What did you think the main purpose of the literature comprehension tutorials and assessment was?
2. How did the tutorials and assessment help to improve your ability to analyse and discuss a paper?
3. How did the tutorials and assessment help you (or not) to understand the assessment process?
4. Did the tutorials make you feel more confident about the assessment? In what way?
5. Do you think the tutorials came at the right time in your degree? When would be the best time to bring these in?
6. Did the tutorials help you understand where the exercise fitted in to your overall degree development and how?
7. Can you tell us some things you learned from the tutorials that have applied or think you will be able to apply in other settings?

Questions regarding Graduate attributes
1. What kinds of things you’re learning about now do you think you will be able to use in your future careers?
2. What, in your mind, are the key graduate attributes a Biomedical Sciences student needs to have gained when they complete their degree?
3. Do you think you’ve had the opportunity to develop any of these attributes so far in your degree—if yes, please give us some examples
4. At the time, did you realise you were developing a graduate attribute?

### Integration of Graduate Attributes Into Biomedical Competency Pyramid

**FIGURE 4:**
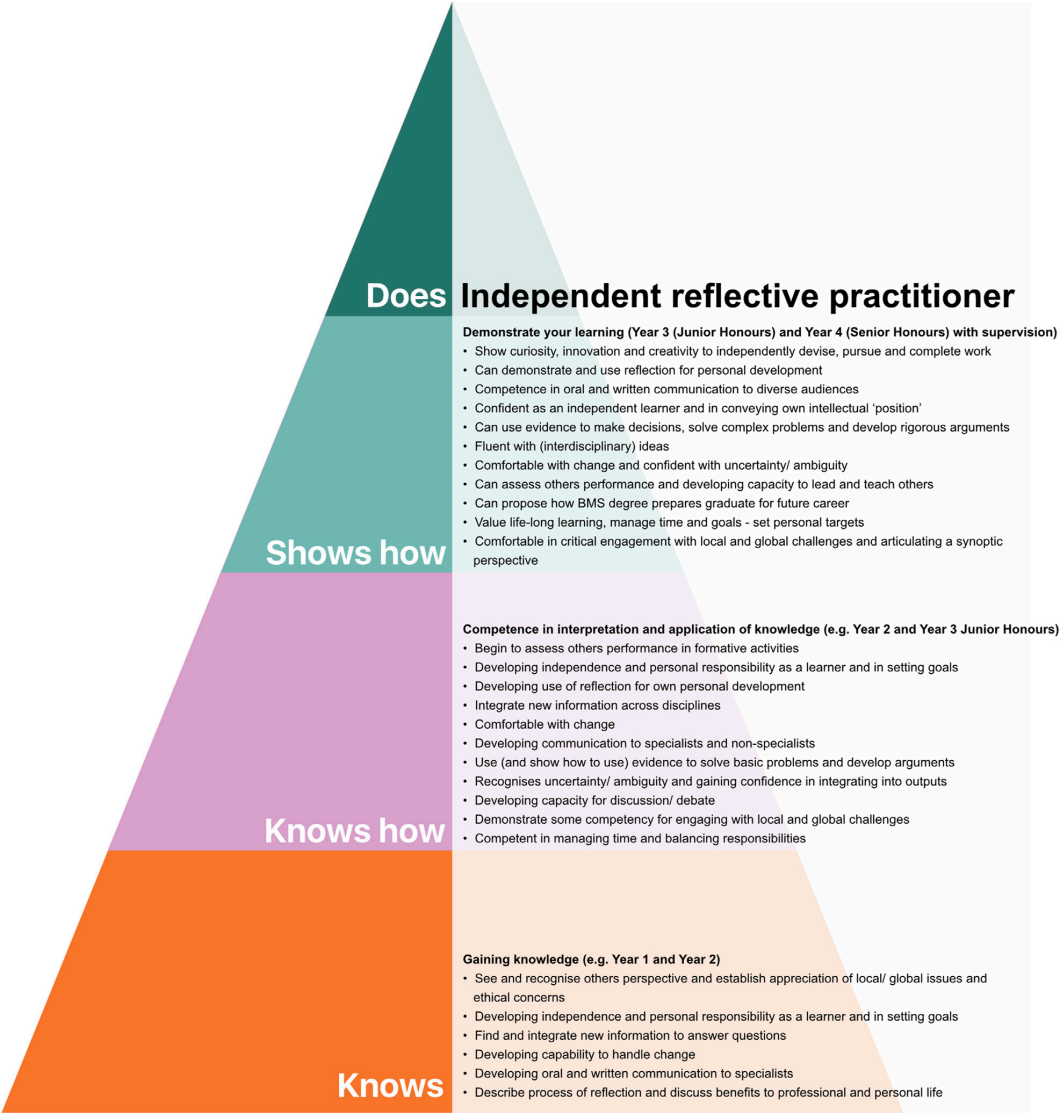
Biomedical Sciences: Undergraduate to Practitioner Graduate Attribute Pyramid. Using Miller’s pyramid as a framework**,** the University of Edinburgh Graduate Attribute Framework, and the Subject Benchmark Statement for Biomedical Scientists (2019) were used to identify and map graduate attribute development from degree entry to graduation.

Having used the BMS competency pyramid (Figure 3) as part of the assessment literacy intervention described here, we sought to develop this aspect further and integrate graduate attributes into a pyramid model. For this, the UoE Graduate Attribute Mindsets and Skills framework [19] and Subject Benchmark Statement for BMS were used as a reference. The graduate attribute pyramid generated during this study is presented in Figure 4.

### Ethical Approval for Study

Ethical approval for both the survey and focus group were obtained from the Social Research Ethics Group (SREG), Deanery of Biomedical Sciences (sub-group of the Research Ethics Committee, School of Health in Social Science, University of Edinburgh).

## Results

### Students Tend to Award Lower Grades Than Faculty

**FIGURE 5:**
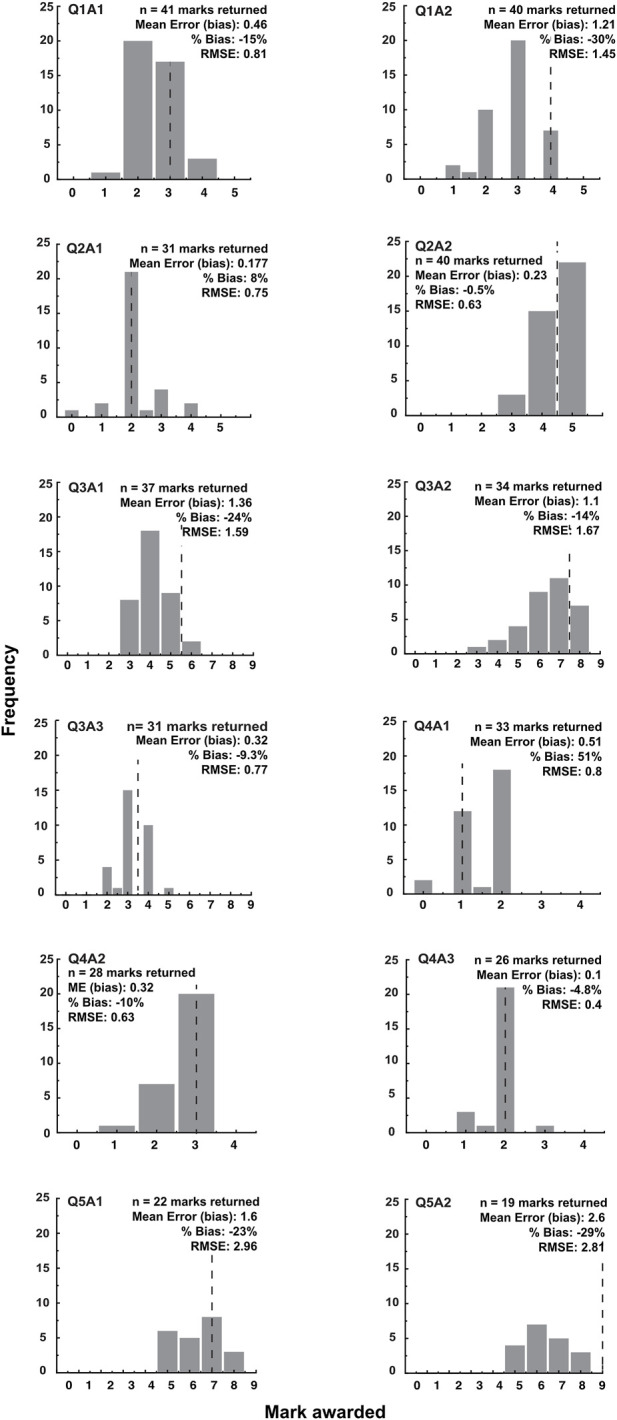
Students tend to award lower marks than faculty. Comparison of student and faculty grades for 5 questions used in formative tutorial 2 of the literature comprehension teaching. Histogram shows frequency of grades returned from 8 tutorials and dotted line represents mean of grades awarded by two independent markers for question. Student (Mean Error) bias was calculated as an average of the difference between each student grade and the recorded faculty grade for each question. Percentage bias as a function of the actual grade for each question was then calculated. The root mean square error (RMSE) was also calculated to reflect the variation of student grades around the faculty grade.

In eight separate literature comprehension tutorial 2 sessions undertaken in 2020, student grades were recorded for 5 questions (12 answers in total). Histograms derived from this data (Figure 5) show variations in the distribution of marks awarded by students for each question. A dotted line indicates the mean mark awarded for the question by two independent faculty markers. Percentage bias for each question is indicated and shows that for 10 out of the 12 answers, students returned lower marks than faculty members. The maximum percentage bias was −30% highlighting that most students had awarded a lower grade than faculty for this question (Q1A2).

### Positive Impact of Assessment Literacy Intervention On Student Confidence in Literature Comprehension Assessment

In 2020, having migrated all teaching of the formative literature comprehension tutorials to an assessment literacy format, our next step was to explore student understanding of their assessment to-date, find out if they were positive about the changes we had implemented and, ultimately, discover if they felt more confident about their upcoming assessment. To achieve this, at the conclusion of the final preparatory tutorials, 186 students across the eight tutorial groups were asked to complete Likert scale questions related to how prepared they felt for their assessment. 159 questionnaires were returned, and the data is presented in Table 1. In brief, students broadly agreed that they had a good understanding of how their assessments were marked (111/159 agreed or strongly agreed) and indicated they consider this an important aspect of their learning. Notably, students indicated the assessment literacy intervention had helped them understand more about different assessment standards (139/159 (87.4%) indicated they agreed or strongly agreed). Related to this, most students agreed or strongly agreed that the tutorials had helped them prepare for their exam [138/159 (86.8%)] and made them feel more confident about communicating their own interpretations and reasoning related to primary research papers [113/159 (71.1%)]. Importantly, 127/159 (79.9%) students indicated that they agreed or strongly agreed that the teaching had made them consider what a reader needs to know. Further, 118/159 (74.2%) students agreed or strongly agreed that the tutorials had helped them evaluate and use data to support their answers to questions. The broadly positive response we received via the targeted tutorial questionnaire was supported and reinforced by later free text comments gathered in the standard Deanery end of course survey (2020):

“I liked the way they were structured. We got to have a practise on our own before the live tutorial. Marking previous answers definitely helped me in understanding how to approach my own answers.”

“It was really nice to learn more about the marking schemes, which helped me better understand the learning outcomes for the assignment and in general the quality and kinds of specific details markers look for in good answers. I was also able to apply the skills I learned in the tutorial sessions to similar assignments in other courses”

“I liked the tutorials as it gave an opportunity to consolidate learning. They also gave an idea of what the Literature Comprehension Assessment would be like, which I found beneficial to help remove any anxiety I had about the assessment.”

### Students Are Aware of Graduate Attributes and Value Their Development

During phase 1 (2019) delivery of our new tutorials, discussions with students as part of our teaching indicated that our assessment literacy approach had not just helped support their engagement with infection-related primary research, it may also have helped facilitate the development of graduate attributes. Amongst other aspects, grading answers of different standards focused students on the logic of their analytical approach, on how they communicated, and encouraged them to reflect on their own work and exercise critical judgement. Given this observation, in 2020 we sought to find out more about student comprehension of graduate attributes and to explore student perceptions of what they had learned from the tutorials. To achieve this, as part of the 2020 end-of-tutorial questionnaire, we integrated several graduate attribute-related questions. To begin, we asked students if they had heard of graduate attributes. Of those who responded (135/159), most (97/135) replied “yes,” whilst 38 had not heard of this term. To follow this up, using Likert scale questions we proceeded to ask students if they valued the development of graduate attributes and if they know when they are developing graduate attributes as part of their degree. Responses to these questions showed students consider the development of graduate attributes a very important aspect of their degree [147/159 (92.5%) agreeing or strongly agreeing]. Notably, 97/159 (61%) of students felt they knew when they were developing graduate attributes as part of their normal degree work with less than 1% unsure when graduate attribute development is occurring.

**FIGURE 6:**
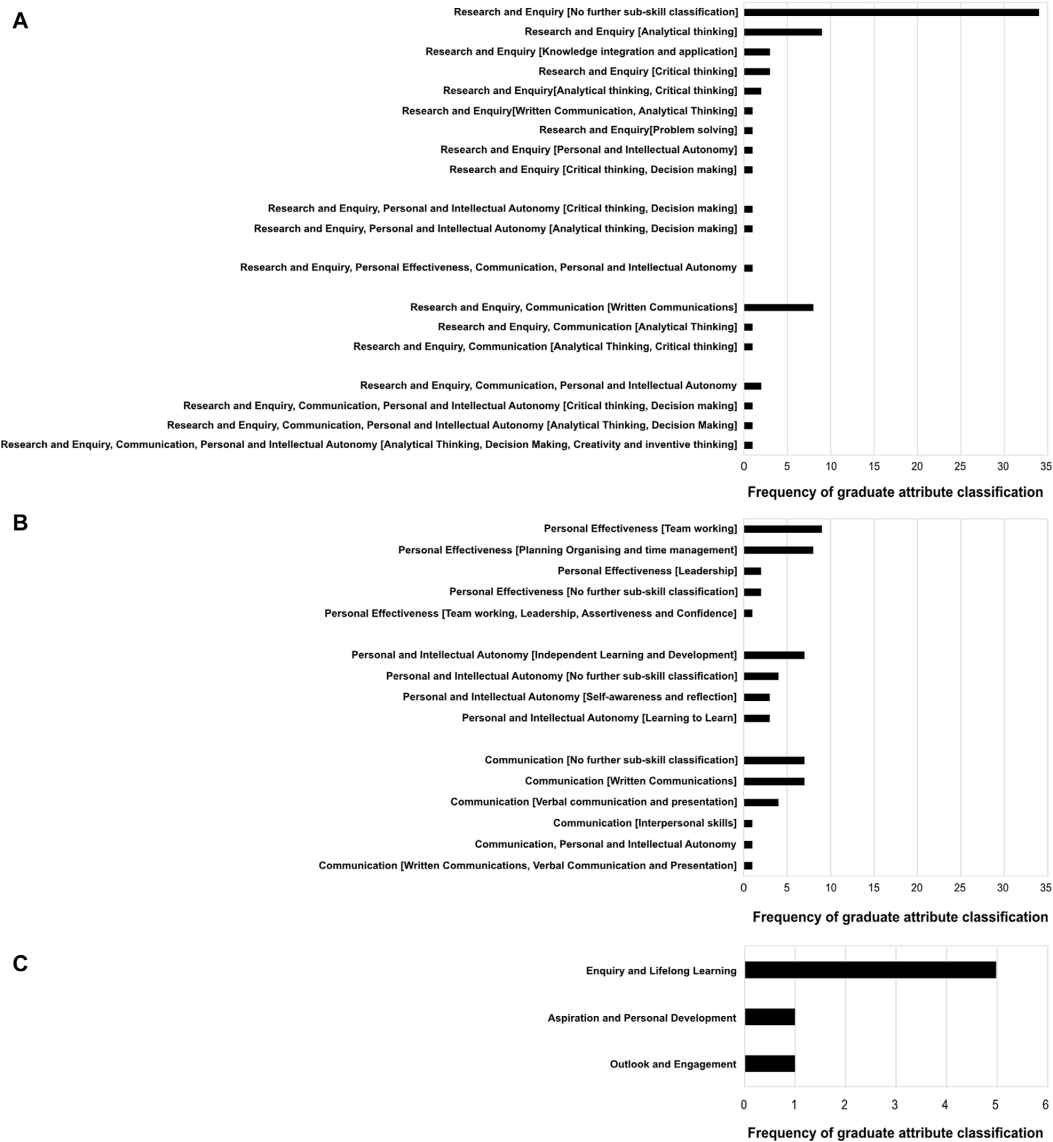
Years 1 and 2 of BMS degree are perceived by students as valuable for the development of graduate attributes related to Research and Enquiry. Year 2 Biomedical Sciences students who had completed the literature comprehension assessment tutorials in 2020 were asked to give examples of graduate attributes already developed as part of their year 1 and 2 studies at the UoE. 115 answers were returned (from 159 questionnaires) as free text. Eighteen were excluded from further analysis as their meaning was unrelated to graduate attributes or considered ambiguous. The remaining statements were then mapped to the UoE Graduate attribute framework according to mindset, skill group and [sub skill group] (indicated in square brackets). Student statements were then grouped according to their mapping classification and group size totals for each classification calculated. Panel **(A)** shows frequencies of statements where classification included “Research and Enquiry.” Panel **(B)** shows frequencies of statements classified as “Personal Effectiveness,” Personal and Intellectual Autonomy’ or “Communication.” Panel **(C)** shows frequency of statements classifiable as related to the mindsets “Enquiry and Lifelong Learning,” “Aspiration and Personal Development” or “Outlook and Engagement.”

To explore student perceptions of graduate attributes further, we proceeded to ask students if they could provide examples (in free text) of graduate attributes they had developed to-date in their degree. 115 answers were returned in response to this question. Responses were variable and ranged from *“How to write a lab report”* to *“Questioning and analysis of myself and the world around me.”* To help us systematically analyse the data, responses were mapped to the UoE graduate attribute framework [19]. Following this mapping, to identify themes, classifications of identical type were grouped and quantitated. The results of this analysis are presented in Figure 6. It is important to note that a small number of responses from students referred to specific degree and/or biomedical domain-related skills that would not typically be defined as graduate attributes. To reduce selection bias, and develop a representative view of the student cohort, the majority of these were retained in our analysis unless meaning was unrelated or ambiguous (e.g., “tutorial skills”). See Supplementary Table S3 for statements excluded from the analysis.

The most notable theme emerging from the student responses was that they identified “Research and Enquiry” as the main area of graduate attribute development in years 1 and 2 of their study (Figure 6A). Under this classification, sub-skills that emerged included “critical thinking,” “analytical thinking,” “knowledge integration and application” and “problem solving.” After “Research and Enquiry,” the remaining skill groups (e.g., “Communication,” “Personal and Intellectual Autonomy” or “Personal effectiveness”) had a similar representation in the data (Figure 6B). Importantly, year 2 BMS students referred to very few attributes that could be classified as related to a “Mindset” as defined in the UoE graduate attribute framework (Figure 6C) [19]. Where a “Mindset” could be applied to a proposed attribute, the most common classification was “Enquiry and Lifelong Learning”. Examples of student statements falling under this classification included “Confidence of how to learn from mistakes,” “Being critical of my own work as well as others” and “Ability to take responsibility for my own learning.” Notably attributes that could be classified as “Outlook and engagement” (2 statements) (“Understanding the relevance of work and its effect on future research” and “Self-motivation”) or “Aspiration and personal development” (1 statement) (“Insight into the qualifications and experience needed to go into a career in academia or research”) were sparsely represented in the data.

**FIGURE 7:**
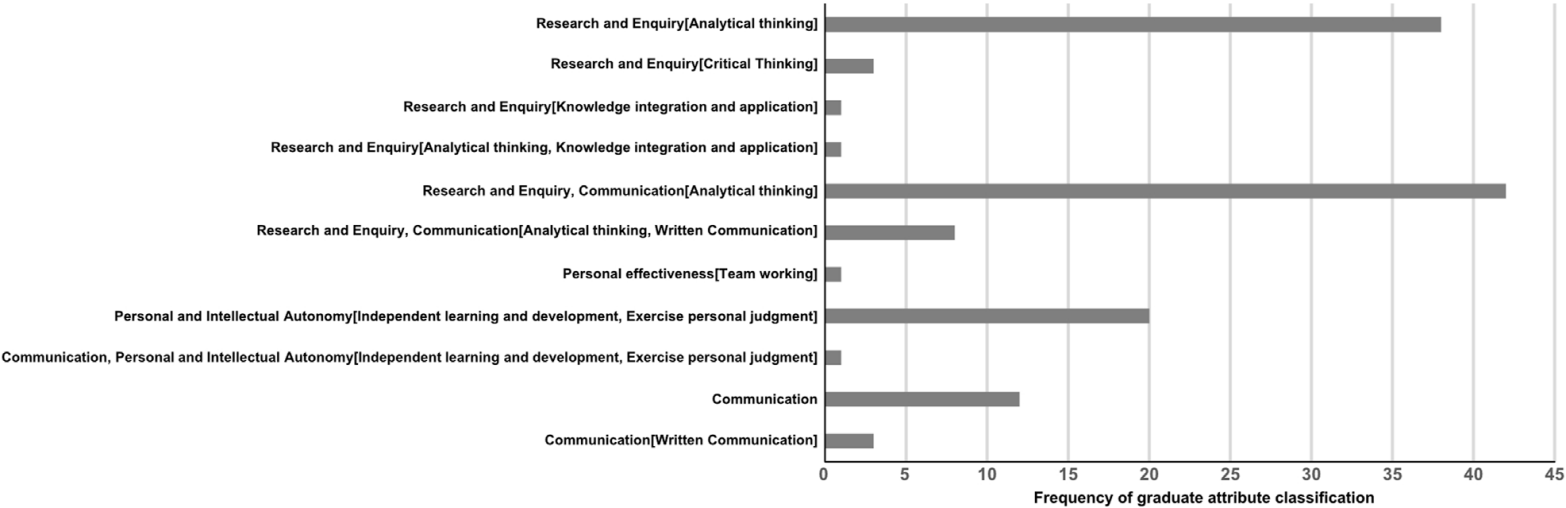
The assessment literacy intervention focused students on the development of graduate attributes rather than domain specific knowledge. Year 2 Biomedical Sciences students who had completed the Literature comprehension assessment tutorials in 2020 were asked to define the most important thing they had learned from the teaching. 142 responses were returned (from 159 questionnaires) as free text. Thirteen responses were excluded from further analysis as the meaning was unclear or considered ambiguous. The remaining statements were then individually mapped to the UoE graduate attributes according to mindset, skill group and, where possible, [sub skill group] (indicated in square brackets). Student statements were then grouped according to their mapping classification and group size totals for each classification calculated.

Given our earlier observation (2019) that students in our assessment literacy tutorials were focussing much of their discussion, questions and learning on the development of broad skills related to graduate attributes, we used our 2020 questionnaire to ask students to state the most important thing they had learned from our teaching. 129 responses to this question were mapped to the UoE graduate attribute framework and themes identified as above (Figure 7). As before, to reduce selection bias, and develop a representative view of the student cohort, the majority of these were retained in our analysis unless meaning was unrelated or ambiguous. See Supplementary Table S4 for statements excluded from the analysis.

Notably, no student responses stated the most important thing they had learned was a specific aspect of the infection-related biology covered in our papers. Almost all responses could be mapped to the graduate attribute framework with a small number excluded from our analysis (e.g., “The kind of questions expected in the exam”). Once again, most student responses (93 (72%) statements classified into this category) could be classified as related to “Research and Enquiry.” Examples of statements grouped into this category include “How to take more from a research paper—understand figures and data and analyse them” and “How to pick out important information and which pieces of data are required to draw meaningful conclusions.” Alongside “Research and Enquiry,” “Communication” was a clear theme evident in the data (66 statements (51%) classified into this category). In this regard, statements such as “To answer questions with adequate detail and to refer to data and figures in my answers” and “How to communicate elements of a scientific paper to others” were classified into this category.

Notably, a clear theme emerging from the statements on important learning outcomes related to “Personal and intellectual autonomy” (21 statements (16.27%) were classified into this category). Specifically, a range of statements indicating enhanced confidence in independent learning and exercising judgement. These included “It was really useful to see an actual mark scheme—gives me a better idea of what you look for” and “How to approach a question because we got to see the marking scheme which made it clearer to me to what the markers are looking for.”

### Long-Term Benefits of Assessment Literacy Literature Comprehension Teaching

The data described above were gathered at the time of (or shortly after) the tutorials and assessment were undertaken. Given the intended function of this teaching is in the development of foundational skills supporting later development (“Knows” and “Knows how”) we wanted to explore how final year students felt this work had influenced their later learning. To achieve this, all 4th year students who had undertaken and completed assessment literacy tutorials (before COVID disruption) in 2nd year (*n* = 186) were invited to contribute to a focus group and four students agreed to participate. When asked what they remembered about the tutorial purpose, student recall of the teaching was variable, however, 3 out of the 4 participants responded with answers that indicated they felt the teaching had been beneficial. For example:

Participant 3:

[in the past] “I was confident with like understanding what the point of the paper was. Just from, you know, abstract and conclusion mainly, but what I found difficult is understanding like how exactly the method was, what exactly did they use this marker for or what was the point of that enzyme. I remember them asking into like very very details of the methods. Which I found quite difficult, but I think it was beneficial 'cause then we actually were forced to learn, to understand how they made up the experiment or how to connect the dots a bit better.”

To develop the discussion, students were then asked if the teaching influenced their understanding of the assessment process. A key theme from answers to this was that students felt the teaching did provide insight into expectations for the assessment. For example:

Participant 1:

“ … the tutorial questions were really quite difficult from what we remember … and it did probably show you how much detail they were expecting … yeah, the tutorials definitely showed you how much in depth they were wanting.”

When asked to consider whether the literature focused tutorials were undertaken at the correct time in their degree, students responded positively. For example,:

Participant 1:

“I think going in that much depth it was probably the right time … I think if someone had said to me in first year, here's some questions on these papers, I would have internally exploded. But at the same time something along those lines, but maybe a bit more basic might have been handy in first year … I think yes, end of second year is probably about right.”

To explore the long-term impact of the teaching, participants were then asked if they thought the tutorials and paper analysis had helped in later years of their degree. Notably, responses to this question were variable and context dependent. One response indicated they felt the teaching had been broadly beneficial, whilst another indicated it was directly relevant to their current work.

Participant 3:

“I think probably unconsciously. I don't think I would particularly think back to the tutorials and think that definitely helped me in what I’m doing now, but I think it was just one of those skills you pick up along the way and you don't even realize that you’ve got it until now you can do it fine.”

Participant 2:

“…my project is a systematic review of technologies … it’s definitely very, very literature understanding based … so for mine it definitely applies”

Finally, having questioned the students on their recollections of the tutorials and their impressions of the benefits, the group were asked “what sorts of things that you’ve picked up along the way during your degree and that you’re doing now in your work [studies] will you be able to apply in whatever you want to do in the future?” Answers were varied but included mention of the benefits of domain specific knowledge as well as a variety of aspects related to graduate attributes (e.g., time-management, communication to varied audiences and a propensity to be more inquisitive).

Participant 3:

“I would say that the degree has made me more inquisitive, so I'm more likely to wonder about things and then want to go and find out more.”

Participant 1:

“I would say I think it's very general as well, but definitely from our experience, just general like essay writing and like writing skills.”

Participant 2:

[Comfortable with] “A multidisciplinary approach”.

## Discussion

In the work described here, we have successfully transitioned an assessment literacy strategy from a vocational veterinary teaching context to a foundational BMS learning activity [13, 18]. As an outcome of this, learning became student-focused and engagement in tutorials was enhanced. Importantly, students reported greater confidence in their understanding of how marks were awarded, the features of a good answer and in preparing for their assessment. An unexpected yet welcome outcome of this approach was that our assessment literacy-based teaching functioned as a vehicle for graduate attribute development within a domain-specific activity. The implications of this observation to our BMS teaching will be discussed further here.

The past 20 years has seen a sometimes-controversial shift in the focus of higher education teaching [20]. Over this period, universities have seen their remit widened and it is now accepted they must develop not just discipline-specific graduates but also provide a general foundation for graduate attributes that enhance employability [21, 22]. This presents several challenges. As Green *et al.* point out, graduate attributes have proven difficult to define and are perceived in a variety of ways by academics [21]. As a result, constructive communication between academics, and between academics and students, regarding graduate attribute development has been hard to achieve [21]. Like many higher education institutions, the UoE has published a graduate attribute statement that serves to establish the generic skills and dispositions students can develop during their degree [19]. A key question is how can the development of graduate attributes be integrated into existing curricula and disciplinary contexts? One response to this has been curriculum mapping—most commonly undertaken for degrees integrating some form of professional accreditation or recognition (e.g., HCPC approved degree programme mapping to Standards of Proficiency for Biomedical Scientists) [9, 23]. Curriculum mapping can be useful in identifying existing graduate attribute development activities that are not addressed in, for example, learning objectives. It can also identify requirements, opportunities, and potential linkages between years in the curriculum. Importantly, once mapping is complete, a key question is how can the teaching and learning environment be adjusted to focus students on the development of graduate attributes in their domain? Notably, whilst assessments can serve to motivate students to engage in learning, recent data suggests the explicit assessment of graduate attributes may be unpopular with students [24]. Focus group analyses revealed students did not think assessment of graduate attributes would serve as an incentive for engagement [24]. Further, some students felt assessment would engender an increased emphasis on marks and may prove to function as a personal affront [24].

At the outset of this study, we aimed to adopt an assessment literacy approach to help students learn how to read, analyse, and communicate their interpretations of primary research papers. On completion of our teaching, feedback from students indicated this strategy moved our teaching away from a teacher- and domain-centric approach and enhanced student confidence and competence in both the process of assessment and literature analysis. In agreement with previous studies, the data presented here show notable variation in the ability of students to accurately grade work. In contrast to previous work, however, where over or under grading was not consistent, in this study students tended to award lower grades than faculty [13]. Exploration of this finding, by further discussion of grade differences with students in tutorials, revealed a key disparity between faculty and student perspective. Students often demonstrate a focus on the concept of losing marks and the presence of a final, definitive conclusion as a key requirement for mark reward. To address the above required that we consider the students ‘metacognitive’ development—how could we facilitate the development of a marker’s perspective in students? We now ensure our approach emphasises that faculty adopt a “positive marking” philosophy—rewarding rather than taking away. We also emphasise the importance of considering the audience, the value of contextual information, and that marks are accumulated through the development of clearly communicated, systematic answers. Students are encouraged to reflect on the needs of the audience and answer questions such as: what was the authors question? What did the authors do? What does the data show? What interpretations and conclusions can be drawn? What do I need to communicate? By providing this process for developing their responses, and engaging students in marking answers following the same logic, the assessment literacy approach can help students focus on how to analyse and develop an answer.

At the conclusion of our 2020 teaching, in contrast to previous years, no student feedback relating to the year of publication of the primary research papers and the relationship between tutorials and lectures was received. We ascribe this to the inclusion of an introductory presentation used to explain the aim of the teaching/assessment and the assessment literacy approach. Notably, students did, however, report enhanced confidence in, and the development of, skills and attributes beyond the domain-specific area (infectious diseases). These attributes could be classified according to the UoE graduate attribute framework as enhanced skills in research and enquiry, communication and, importantly, independent learning and exercising personal judgement [19]. By engaging students with standards and expectations, evidence to-date, therefore, suggests assessment literacy can facilitate the engagement with, and development of, graduate attributes.

As an integral part of this work, Miller’s pyramid was adapted to show BMS competency development from degree entry to practitioner [18]. This helped us communicate to students where their literature comprehension teaching and assessment fitted into overall BMS competency development. In doing so, it helped us address the need for a “transparent” curriculum and provide students with the opportunity to work towards “declared” objectives and plan for future skill development [25]. Overall, we view this representation as dynamic and envisage it will evolve over time as we receive input from colleagues and other stakeholders (see limitations below). Importantly, to extend this work the pyramid approach facilitated the systematic mapping of UoE graduate attributes to BMS competency development - allowing us to conceptualise graduate attributes in a specific domain context (Figure 4). A future objective is to test how this helps us to convey to the students how graduate attribute development can evolve over the degree and what can be expected at different levels.

Importantly, the development and use of the BMS competency pyramid highlighted several key issues. The work described here indicates a requirement for a systematic analysis of our entire BMS curriculum with the aim of identifying requirements and opportunities for graduate attribute development and assessment embedded within or alongside current teaching, learning and assessment activities. In this regard, our work agrees with recent findings showing limited evidence for specific educational approaches driving the systematic development of graduate attributes in UK undergraduate degrees [26]. Several models for curriculum and graduate attribute mapping exist and the activity will have to complement or be part of an ongoing curriculum transformation programme at the UoE [22, 23, 27, 28]. Given our data emphasising the importance students place on graduate attribute development, it would seem prudent that this process is undertaken in partnership with students [29].

Use of the competency pyramid and parallel analysis of student questionnaire responses emphasised a focus on student attribute development related to Research and Enquiry in years 1 and 2 of the BMS degree. This was expected given an early teaching focus on formative activities enabling academic competency and a transition to university. Importantly, analysis of year 2 student questionnaire data revealed a focus on graduate attributes defined as “skills” by the UoE graduate attribute framework [19]. These data, and the variable responses we obtained regarding the long-term impact of competency and graduate attribute development in our focus group, highlight an opportunity for use of assessment literacy throughout our curriculum. As a next step, we plan to explore the use of assessment literacy and regular engagement with the competency/graduate attribute pyramid model in all years to help students acknowledge and reflect on their development. In doing so, they may recognise when changes in, for example, their outlook or mindset occur as they progress through the degree. In this regard, it was notable that in our focus group, one student did remark that they were more “inquisitive” at the conclusion of their studies. Evidence on undergraduate mindset development is limited and studies that have emerged suggest undergraduates do not change mindset over time [30]. Of some concern, are studies that indicate STEM students develop an increasingly fixed mindset as they progress through their studies [31]. A key future objective for our work, therefore, is to explore how we can use assessment literacy and our competency/graduate attribute model throughout the curriculum to help students set and importantly achieve objectives that demonstrate development and promote “growth” mindsets enabling them to take on challenges and achieve success [30]. Notably, a recent study described peer interaction—integral to our assessment literacy approach—as influential in determining student mindsets [30]. Whilst it was not a focus of the work described here, involving students in discussion of assessment, and reflecting on how it has impacted their development, could also be useful as a means of gathering valuable additional insight into their perspective as partners in the assessment process; in particular in relation to key aspects such as inclusivity and the impact assessment has on student wellbeing [32, 33].

To conclude, as several authors have noted, graduate attributes are not generic and their definition, and how they are perceived, differs between disciplines [21, 34, 35]. To address this, it has been proposed that teaching processes make it clear how aspects of a degree (including assessments) contribute to graduate attribute development. This will help students recognise how their study might prepare them for later work [26]. Models developed to enhance assessment literacy may help to achieve this by engaging students with process, purpose, application of standards and expectations. In doing so, they may be used to enhance skills, aptitudes and dispositions enabling parallel academic achievement and transition to the workplace.

### Limitations

There are several limitations to consider when interpreting these data and drawing conclusions. Firstly, the data gathered here was from a single course, at a single institution. Whilst the UoE BMS student cohort is typically drawn from a diverse range of cultural and educational backgrounds, we cannot predict that the findings will be generalisable to other contexts. The study could be strengthened by replication with a more representative sample of undergraduates.

In relation to the study design, a clear limitation relates to the size and composition of our focus group. Students volunteered to participate in this exercise and, therefore, represent a very limited portion of potential respondents. In both the questionnaires and the focus group, we have captured self-reported responses to our teaching. Additionally, in the case of the focus groups, students reported retrospectively. As a result, our data are prone to recall bias and other cognitive biases and may not be representative of the wider student population.

In the comparison of the student and faculty grades, two members of faculty had originally marked the answers analysed in the tutorials. As such, it was not possible to apply statistical testing to enhance the validity of our conclusions in this regard. The study could be strengthened by the addition of further faculty markers. Not only would this strengthen the statistical analysis, but we also anticipate a wider faculty contribution would generate valuable discourse re. what is, and should be, rewarded in an assessment.

At the outset of this project, a key aim was to evaluate the year-on-year effect of the assessment literacy intervention on overall class grades. Ultimately, this was not possible due to changes in delivery of the assessment in response to the COVID pandemic. In 2020, the exam moved from a 90-min invigilated format to an online assessment undertaken over a 24 h period. For both academic and practical reasons, this online delivery method has been retained and, with no like-for-like comparison possible, we have not sought to directly test whether our intervention had a positive effect on cohort grades. Further studies to directly test the impact of assessment literacy intervention are required, however, the similarity of adjacent cohorts cannot be assumed.

In relation to our data analysis, a methodological limitation relates to the mapping of respondent data to the graduate attribute framework. Every effort was made to undertake this in a systematic manner and response classifications were agreed between authors. Notably, however, an absence of, for example, a controlled vocabulary means this aspect of the study may be subject to bias.

The work described here was undertaken using existing definitions of graduate attributes as defined in the UoE graduate attribute framework and described in the literature. This may be considered a limitation, and future studies would benefit from more active dialog with employers with the aim of defining specific competencies and attributes considered desirable in the graduate workplace. This input would be valuable to future curriculum development.

## Summary Table

### What Is Known About This Subject

• Biomedical Sciences degrees must provide domain specific learning and prepare graduates for work and life after their studies.
• Assessment literacy based teaching enables students to use an appropriate, relevant method for any given assessment task.
• An absence of assessment literacy can impede an individual’s capacity to learn and can limit inclusivity, equity, and participation in higher education.

### What This Paper Adds

• Assessment literacy teaching enhanced student engagement in tutorials.
• Assessment literacy teaching improved confidence in student understanding of standards and in preparation for an assessment.
• Assessment literacy teaching also facilitated graduate attribute development within a domain-specific activity.

## Concluding Statement

This work represents an advance in biomedical science because it shows that assessment literacy teaching in a BMS degree may be used to enhance skills, aptitudes and dispositions enabling parallel academic achievement and transition to the workplace.

## Data Availability

The datasets presented in this article are not readily available per ethics approval. Requests to access the datasets should be directed to KR, kevin.robertson@ed.ac.uk.
