# Supplementary material for: Embedding Assessment Literacy Can Enhance Graduate Attribute Development in a Biomedical Sciences Curriculum
Source: Br J Biomed Sci. 2024 May 24;81:12229. doi: 10.3389/bjbs.2024.12229 (PMC11160838; doi:10.3389/bjbs.2024.12229)
Supplement: Supplementary file 1 [file DataSheet2.pdf]

Figure S2

A.

Explain in your own words how the data in Figure 2 expands on the results in Figure 1? [8 marks]

Describe general features of Fig 1 and then concisely describe the main findings – good!

Faculty Mark

Answer 1:

1 B

"In Figure 1 the authors show how adding in more bacteria to the well (x-axis) affected percentage binding (y-axis) for phagocytes from different strains of mice and different places in the body (peritoneum or lungs).

1 A 1 C

The main finding in figure 1 is that when the authors added more bacteria to the cells, more bacteria were bound. The results were similar for all the peritoneal macrophages – the alveolar macrophages seemed to bind less bacteria overall. 1 C

5:6

1 D

In Figure 2 the authors did the same experiment with more types of phagocytes from Guinea-pig and Human and saw similar results.

1 G

Therefore, Figure 2 extends Figure 1 by showing that phagocytes from species other than mouse show similar ability to bind bacteria."

Concise summary of findings in Fig 2 Rounded off with a statement answering the question.

Markers notes:
Good systematic answer BUT **requires data description** for final marks

B.

Marking guidelines:
It is expected that the students will employ a systematic approach to answering this question and briefly describe the data/conclusions from both Figures 1 and 2 in their answer.

Figure 1

a. Authors asked the question: Do macrophages from different mouse strains and anatomical locations have a quantifiable difference in ability to bind *S. albus*? **(1)**
b. Figure 1. is a graph showing log bacterial concentration on the X axis and percentage macrophages bound on the Y axis. The data are the mean of 3 experiments and error bars show the standard error of the mean. **(1)**
c. From this data, it may be concluded that percentage binding was greater at higher concentration of bacteria, peritoneal macrophages appear to bind more bacteria than alveolar macrophages and macrophages from different strains bound bacteria to a similar extent. **(2)**

Figure 2

d. Figure 2. is a graph of same format as Fig1. Shows data for Guinea pig macrophages, neutrophils and eosinophils and Human monocytes, neutrophils and eosinophils. **(1)**
e. Overall, percentage binding is lower (max 45% in GP alveolar macrophages compared to 80 to 95% in mouse experiment).
f. With exception of GP neutrophils, broad trend of increased bacterial binding at higher concentrations of bacteria is also observed. **(1)**
g. Therefore, Figure 2 extends Figure 1 by showing that the observed binding of bacteria by mouse macrophages is conserved in phagocytes from 2 further species (GP and Human). **(1)**

Figure S2. Example of literature comprehension question, answer and marking guidelines used in Tutorial 2.

(A) Shows question (in box) referring to primary research paper which was the subject of the tutorial: Glass E, Stewart J, Weir D. Presence of bacterial binding' lectin-like' receptors on phagocytes. Immunology. 1981;44(3):529.

Students were asked to answer this question themselves before the tutorial. In the teaching session, students graded 3 authentic answers (of the type shown below question). Having graded the authentic answers, faculty marker comments (red) and marks awarded (red circles) were revealed and discussed. Letters show marking guideline with which mark was associated.

(B) Shows marking guidelines for grading of question.
