# Supplementary material for: Embedding Assessment Literacy Can Enhance Graduate Attribute Development in a Biomedical Sciences Curriculum
Source: Br J Biomed Sci. 2024 May 24;81:12229. doi: 10.3389/bjbs.2024.12229 (PMC11160838; doi:10.3389/bjbs.2024.12229)
Supplement: Supplementary file 2 [file Table4.pdf]

Table S4. Free text responses to the statement "The most important thing I learned in the MII2 Literature comprehension tutorials is…" that were excluded from the analysis.

"The kind of answers expected in the exam"
"What is expected in the exam"
"A good marking scheme, helpful as usually there are no past paper answers online"
"How the exam is set up - I feel extremely more prepared for the literature comprehension exam now."
"Practicing a procedure when reading a paper."
"How to gain marks in long-style questions without deviating from the question"
"How thoroughly the questions have to be answered"
"The standard of marking at this level of university"
"I know what to expect in the exam"
"When describing data and all relevant details, so add everything in whole figure on table"
"Quality over quantity"
"Quality over quantity in answers"
"Quality over quantity"
