# Supplementary material for: Embedding Assessment Literacy Can Enhance Graduate Attribute Development in a Biomedical Sciences Curriculum
Source: Br J Biomed Sci. 2024 May 24;81:12229. doi: 10.3389/bjbs.2024.12229 (PMC11160838; doi:10.3389/bjbs.2024.12229)
Supplement: Supplementary file 3 [file DataSheet4.PDF]

Embedding assessment literacy can enhance graduate attribute development in a Biomedical Sciences curriculum

Authors
Kevin A. Robertson, Kirsty J. Hughes, Susan M. Rhind

Supplementary Video

Pre-recorded presentation provided to students before their tutorials. Presentation was designed to (a) introduce the purpose of the literature comprehension assessment and its function in wider student development and (b) address frequently asked questions about the teaching material.

https://media.ed.ac.uk/media/Introduction+to+Literature+Comprehension+Teaching+and+Assessment/1_sc2nwt6q
