# Supplementary material for: Embedding Assessment Literacy Can Enhance Graduate Attribute Development in a Biomedical Sciences Curriculum
Source: Br J Biomed Sci. 2024 May 24;81:12229. doi: 10.3389/bjbs.2024.12229 (PMC11160838; doi:10.3389/bjbs.2024.12229)
Supplement: Supplementary file 4 [file Table1.pdf]

Table S1. Course level learning outcomes for the year 2 course UoE Biomedical Sciences course Microorganisms, Infection, and Immunity 2. Course level learning outcome 4 refers to the teaching and assessment integral to the Literature Comprehension Assessment.

**By the end of the course, students should be able to:**	
**1**	Describe the structural organisation, metabolism, growth processes and genetics of microorganisms. Explain how pathogen structure and physiology relates to infection and survival within the host. Describe mechanisms by which infection can lead to disease and immune pathology, using selected examples of microorganisms where appropriate.
**2**	Describe the functions and characteristics of the innate and adaptive arms of the immune system. Explain the roles of the key innate and adaptive immune cells, and how they work together to recognize, respond to, and kill pathogens. Provide an overview of how uncontrolled immune responses can lead to disease and immune-mediated pathology.
**3**	Describe how immune-related (e.g., vaccination) and non-immune (e.g., drug treatments, hygiene) approaches can be used to control infection. Explain how an immune component (antibodies) can be generated and applied as a tool for experimental research or for the therapeutic treatment of diseases.
**4**	Extract, summarise, and interpret information contained within selected primary scientific research papers pertaining to the areas of infection and immunity.
**5**	Perform practical procedures to produce accurate results, explain the theoretical basis of the techniques employed, integrate information from lectures and practicals to interpret experimental data and answer questions related to the implications of their work in a wider context.
