# Supplementary material for: Embedding Assessment Literacy Can Enhance Graduate Attribute Development in a Biomedical Sciences Curriculum
Source: Br J Biomed Sci. 2024 May 24;81:12229. doi: 10.3389/bjbs.2024.12229 (PMC11160838; doi:10.3389/bjbs.2024.12229)
Supplement: Supplementary file 5 [file Table2.pdf]

Table S2. Deanery-wide end of course survey questions (2019) used to gather feedback on Literature comprehension tutorials and assessment.

**Likert scale questions**
Tutorials were interesting, relevant, and useful.
My tutor was knowledgeable and helpful.
The tutorials helped me to learn how to read scientific literature.
If you have received feedback from your Literature Comprehension Test, the feedback received was useful.
The Literature Comprehension tutorials were well organised.
The learning aims of the Literature Comprehension tutorials were clear.
The Literature Comprehension tutorial 2 session helped me understand more about different standards in assessment.
The Literature Comprehension tutorial 2 session helped me better understand how to prepare for the Literature Comprehension exam.
**Free text questions**
Any comments on the tutorials - e.g., what did you like/dislike about them, could they be improved?
