# Supplementary material for: Embedding Assessment Literacy Can Enhance Graduate Attribute Development in a Biomedical Sciences Curriculum
Source: Br J Biomed Sci. 2024 May 24;81:12229. doi: 10.3389/bjbs.2024.12229 (PMC11160838; doi:10.3389/bjbs.2024.12229)
Supplement: Supplementary file 6 [file DataSheet3.PDF]

Figure S3

UoE Graduate Attribute Framework

Graduate attributes are skills and abilities, attitudes and approaches demonstrated by graduates of a higher education institution

Graduate attributes determine how a graduate approaches learning and knowledge, personal development, and the world around them

Mean different things in different contexts and disciplines

Developed through meaningful experiences and the processes of learning and reflection

Are unique to every student

Mindsets

Our usual ways of thinking about something and our attitudes towards it. Mindsets evolve and can change with time and experiences, they influence our behaviours, and they are shaped by our individual values

Enquiry and Lifelong learning

UoE graduates seek personal and academic learning that makes a positive difference to themselves and to the world around them. Inspired by their exposure to world-leading research, they are innovative and lifelong learners.

Aspiration and Personal Development

UoE graduates draw on their initiative and experience to expand and fulfil their potential. Making the most of a confident and reflective approach, they take personal responsibility for pursuing their goals and opportunities to grow.

Outlook and Engagement

UoE graduates draw on the quality, depth and breadth of their experiences to engage with the communities and world around them. With an informed international perspective, they seek to contribute positively, ethically and respectfully.

Skills

Abilities that allow us to perform a task and enable our actions. Our skills get stronger and more flexible through using and reflecting on them in different experiences.

Research and Enquiry

UoE graduates use their highly-developed skills in research and enquiry to identify and creatively tackle problems, and to seek out opportunities for learning.

Skills include: problem solving, analytical thinking, critical thinking, knowledge integration and application, independent Research,

Personal and Intellectual autonomy

UoE graduates critically evaluate ideas, evidence and experiences from an open-minded and reasoned

Personal Effectiveness

UoE graduates are effective and proactive individuals, skilled in influencing positively and adapting to new situations with sensitivity and integrity.

Communication

UoE graduates use skilled communication to enhance their understanding of a topic or context and to engage effectively with others.

Figure S3. Schematic summarising key features of the University of Edinburgh Graduate Attributes Framework. In brief, the UoE Graduate Attribute framework serves to define what graduate attributes are and illustrate how they may manifest themselves in different ways depending on the individual (19). The framework begins by classifying graduate attributes as being either Mindsets or Skills. Mindsets or Skills are then further subclassified into related areas such as ‘Outlook and Engagement’ or ‘Research and Enquiry’. Finally, the framework describes the GA capabilities of a UoE graduate and offers examples of how each Skill or Mindset type can be demonstrated (19).
