# Supplementary material for: Embedding Assessment Literacy Can Enhance Graduate Attribute Development in a Biomedical Sciences Curriculum
Source: Br J Biomed Sci. 2024 May 24;81:12229. doi: 10.3389/bjbs.2024.12229 (PMC11160838; doi:10.3389/bjbs.2024.12229)
Supplement: Supplementary file 7 [file Table3.pdf]

Table S3. Free text responses to the request “Can you give examples of graduate attributes you think you have already developed as part of your studies at the University of Edinburgh? that were excluded from the mapping and analysis.

“Tutorial skills”
“Technical scientific skills”
“Skills and knowledge required for non-research related lab work”
“Understand information obtained from journals/lectures and apply it - tutorials/exams/coursework/practical”
“Academic discussion”
“Becoming more comfortable with scientific literature”
“Experimental skills – practical”
“I developed the practical skills”
“Lab experience”
“Lab skills”
“Lab techniques”
“Lab work”
“Many different lab techniques during practical work”
“Practical skills”
“Understanding how to correctly structure answers in the exam to get marks through peer-review and looking at marking scheme”
“Knowledge in my degree area”
“Some courses require extensive reading so efficient method to grab key points from a lot of reading”
“Knowledge related to everyday life”
