# Supplementary material for: Embedding Assessment Literacy Can Enhance Graduate Attribute Development in a Biomedical Sciences Curriculum
Source: Br J Biomed Sci. 2024 May 24;81:12229. doi: 10.3389/bjbs.2024.12229 (PMC11160838; doi:10.3389/bjbs.2024.12229)
Supplement: Supplementary file 8 [file DataSheet1.PDF]

Figure S1

A.

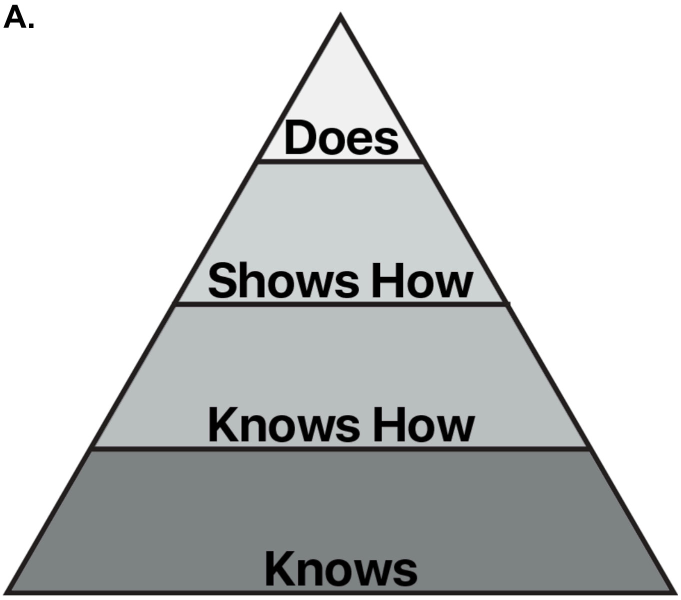

B.

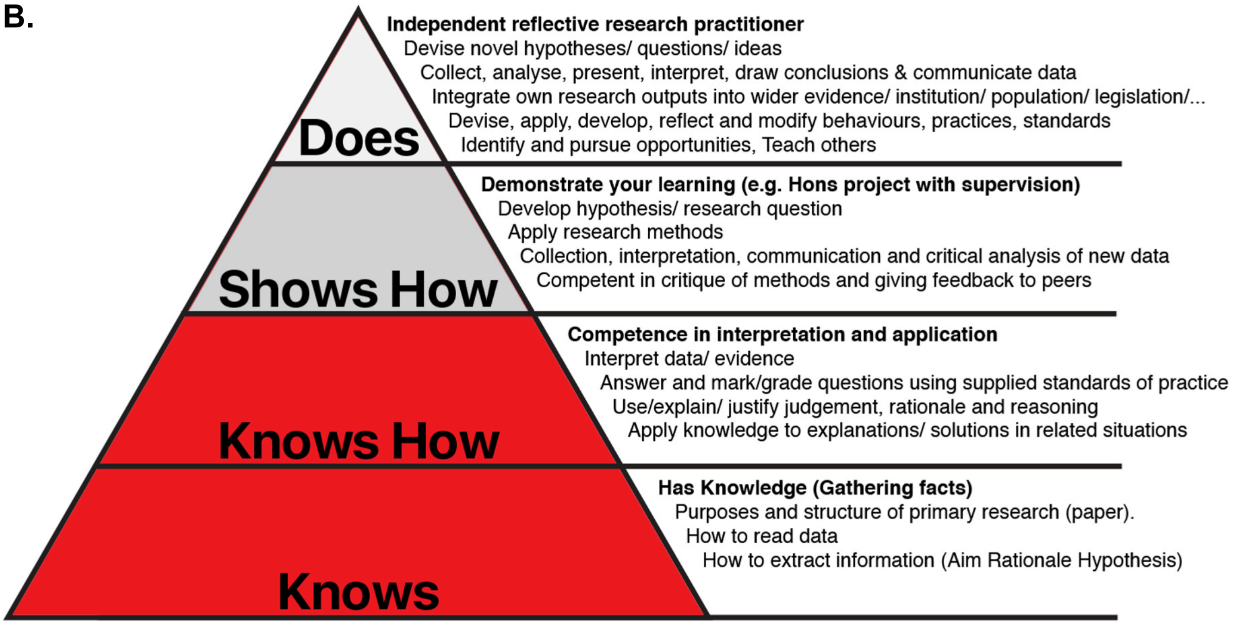

Figure S1. Adaptation of Miller's pyramid to integrate BMS competencies.

Panel A shows Miller's pyramid where the cognitive levels knows and knows how (knowledge and application of knowledge respectively) serve as a foundation for application of knowledge (shows how) and, ultimately, the application of learning as a practitioner (does).

Panel B shows a preliminary mapping of BMS competencies related to literature comprehension to Miller's pyramid levels. The areas marked in red emphasise the levels which students are developing as part of the year 2 assessment.
